# High-resolution DNA quadruplex structure containing all the A-, G-, C-, T-tetrads

**DOI:** 10.1093/nar/gky902

**Published:** 2018-10-04

**Authors:** Hehua Liu, Rui Wang, Xiang Yu, Fusheng Shen, Wenxian Lan, Phensinee Haruehanroengra, Qingqing Yao, Jing Zhang, Yiqing Chen, Suhua Li, Baixing Wu, Lina Zheng, Jinbiao Ma, Jinzhong Lin, Chunyang Cao, Jixi Li, Jia Sheng, Jianhua Gan

**Affiliations:** 1 State Key Laboratory of Genetic Engineering, Collaborative Innovation Center of Genetics and Development, Department of Physiology and Biophysics, School of Life Sciences, Fudan University, Shanghai 200433, China; 2 State Key Laboratory of Genetic Engineering, Collaborative Innovation Center of Genetics and Development, Department of Biochemistry, School of Life Sciences, Fudan University, Shanghai 200433, China; 3 Department of Chemistry and The RNA Institute, University at Albany, State University of New York, Albany, NY 12222, USA; 4 State Key Laboratory of Bioorganic and Natural Product Chemistry, Center for Excellence in Molecular Synthesis, Shanghai Institute of Organic Chemistry, Shanghai 200032, China; 5 Department of Neurology, Huashan Hospital, Fudan University, Shanghai 200040, China

## Abstract

DNA can form diverse structures, which predefine their physiological functions. Besides duplexes that carry the genetic information, quadruplexes are the most well-studied DNA structures. In addition to their important roles in recombination, replication, transcription and translation, DNA quadruplexes have also been applied as diagnostic aptamers and antidisease therapeutics. Herein we further expand the sequence and structure complexity of DNA quadruplex by presenting a high-resolution crystal structure of DNA1 (5′-AGAGAGATGGGTGCGTT-3′). This is the first quadruplex structure that contains all the internal A-, G-, C-, T-tetrads, A:T:A:T tetrads and bulged nucleotides in one single structure; as revealed by site-specific mutagenesis and biophysical studies, the central ATGGG motif plays important role in the quadruplex formation. Interestingly, our structure also provides great new insights into cation recognition, including the first-time reported Pb^2+^, by tetrad structures.

## INTRODUCTION

DNA is highly dynamic biomolecule that can adopt diverse structural conformations including duplexes, triplexes, i-motifs, quadruplexes and other multistranded architectures (1–5). Although the canonical Watson–Crick paired duplexes play major roles in genetic inheritance and gene expression, the structural polymorphism has been associated with many different biological functions of DNA. The most well-studied alternative DNA structures are G-quadruplexes (G4), the four-stranded columnar structures formed by G-rich sequences. Folding of G4 structures is normally stabilized by the stacking of multiple Hoogsteen-hydrogen-bonded G-tetrads and the electrostatic interactions between the guanines and the cations residing in the center of the tetrads. G4 structures are polymorphic and can be intramolecular, bimolecular or tetramolecular with strands parallelly and/or antiparallelly oriented (6–9). Besides the regular right-handed conformation, recent studies showed that G4 can also adopt left-handed conformation (10). The G4-forming sequences have been identified in numerous regions of human genome (11,12), including chromosomal telomeres and many gene promoters, which play important roles in DNA recombination (13), replication (14,15), transcription (16), translation (17) and many other critical biological processes. The discoveries of these sequences have led to significant interest in finding ways to control or modulate the G4 formation. In addition to certain DNA sequences, RNA and other artificial oligonucleotides, such as LNA and PNA (locked and peptide nucleic acids), can also form G4 structures (18–21). These structures have been extensively applied as diagnostic aptamers (22), antidisease therapeutics (23–26), as well as unique building blocks in nucleic acid nanotechnology (27).

The functional importance of G4 has led to extensive biophysical and structural studies, which revealed the great diversity and complexity in quadruplex-forming sequences and their conformations. In addition to the regular G-tetrads, non-G-tetrads including T-, U-, A-, C-tetrads and G:A:G:A, G:C:G:C, T:A:T:A tetrads have been observed in several quadruplex structures of DNA or RNA (6,28–36). Conformations of many non-G-tetrads have also been theoretically predicted (37,38). The non-G tetrad structures could be connected with G-tetrads by various internal loops or bulged nucleotides in the truncated sequences of natural human telomeres, implying their potential biological relevance. Besides the tetrads composition and connection, the diversity of quadruplex structures is further expanded by the ions bound in the center of the tetrad. Various monovalent cations, such as K^+^, Na^+^, NH_4_^+^, Li^+^, Cs^+^, Rb^+^ and Tl^+^ can facilitate the quadruplex formation; binding of the most common K^+^ and Na^+^ cations by the tetrads has been confirmed by many crystal and nuclear magnetic resonance (NMR) structures. Besides monovalent cations, structural evidence and *in vitro* assays also showed that some divalent cations such as Ca^2+^, Ba^2+^, Hg^2+^ and Pb^2+^ can also affect the assembly and functions of the quadruplex structures (39–44). For example, Pb^2+^ can switch the active K^+^-coupled quadruplex structure of the peroxidase-like DNAzyme into an inactive but more stable quadruplex structure, which may play certain role in the toxicity of Pb^2+^ in human. However, due to the lacking of high-resolution crystal structures, the detailed binding conformations of these divalent cations remain poorly understood.

**Figure 1.**
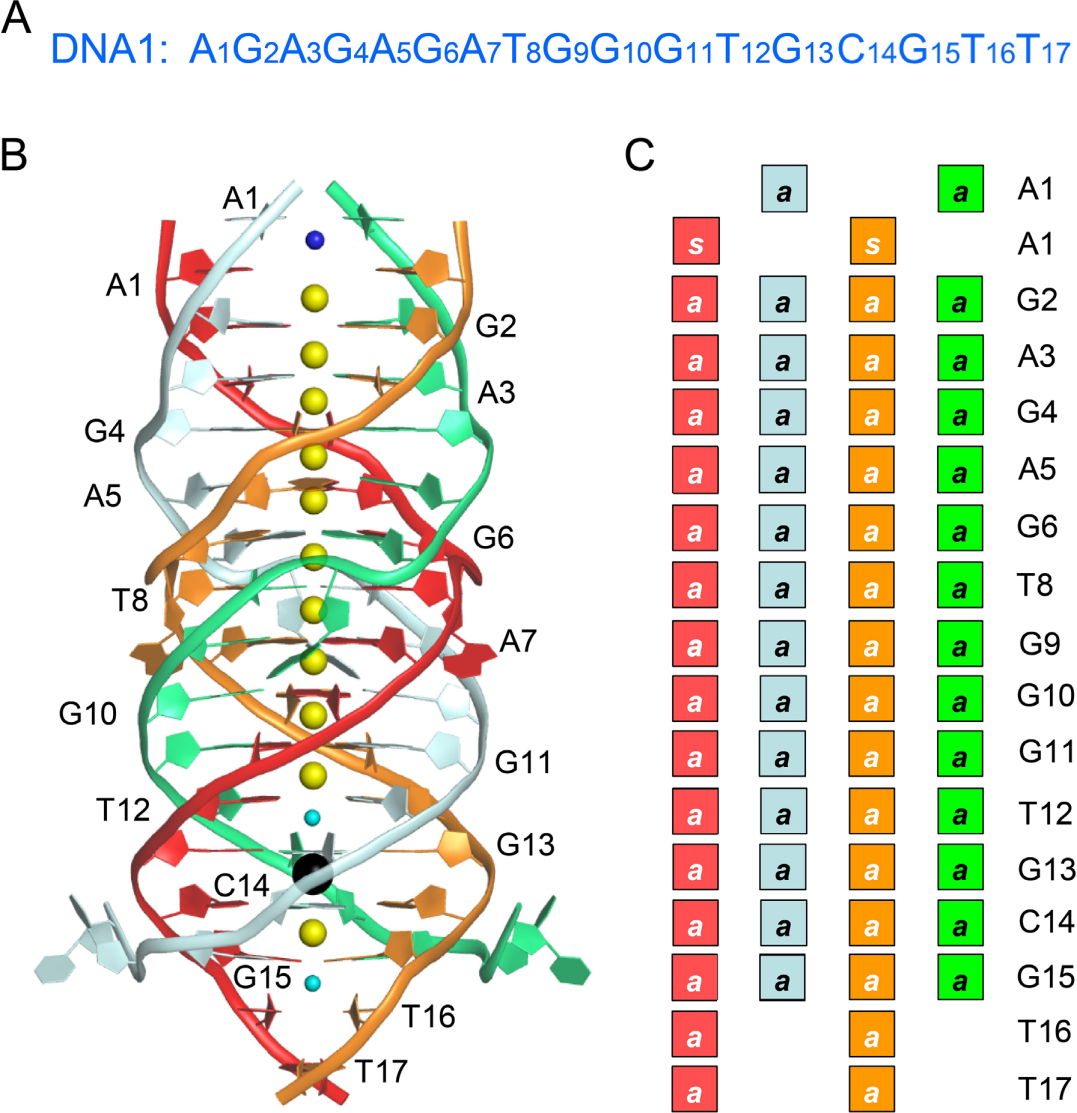
Sequence and overall structure of DNA1. (**A**) Detailed sequence of DNA1. (**B**) Overall structure of DNA1 quadruplex. The DNAs are shown as cartoon-and-ring mode with the four strands colored in cyan, orange, green and red, respectively. The ions are shown as spheres; Li^+^, NH_4_^+^, Na^+^ and Pb^2+^ are colored in cyan, blue, yellow and black, respectively. (**C**) A schematic view showing the glycosidic assignments of each individual nucleotides. *s* and *a* refer to the *syn* and *anti* conformation around the glycosol bond, respectively.

Herein we further expand the structure and sequence complexity of DNA four-stranded architecture by presenting a high-resolution crystal structure of DNA1 (5′- AGAGAGATGGGTGCGTT-3′). This is the first DNA structure that contains all the internal A-, G-, C-, T-tetrads, A:T:A:T tetrads, bulged nucleotides and multiple metal ions including Li^+^, NH_4_^+^, Na^+^ and Pb^2+^ in one single structure (Figure 1). The central residues form one unique kink and 13 stacking tetrads, which are the longest tetrad layers formed within one quadruplex available to date. Our site-specific mutagenesis and biophysical studies confirm that the central ATGGG motif is important for the folding of the structure. The DNA1 sequence is very different from the commonly used G3-L1-G3-L2-G3-L3-G3 one for identifying potential G4 structures in bioinformatic studies (45). Interestingly, via A:T:A:T tetrad formation of the terminal adenine and thymine residues, DNA1 quadruplex can assemble into nanowire-like structure in the crystal lattice.

## MATERIALS AND METHODS

### Crystallization and data collection

DNA1 utilized in the crystallization studies was purchased from the Shanghai GENERAY Biotech Co., Ltd. The crystallization sample was prepared at room temperature by mixing DNA1, 8–17 DNAzyme and PbCl_2_, which were dissolved in ddH_2_O; the final concentrations of DNA1, 8–17 DNAzyme and PbCl_2_ are all 0.5 mM. The crystallization conditions were identified at 16°C using the Gryphon crystallization robot system from Art Robbins Instrument company and crystallization kits from Hampton Research company. The final crystallization conditions are composed of 100 mM CAPS-NaOH pH 10.5, 200 mM Li_2_SO_4_ and 2.0 M (NH_4_)_2_SO_4_; the droplet contains 0.3 μl DNA sample and 0.3 μl crystallization buffer. The growth of the DNA1 crystals was very slow; it took more than 9 months for the crystals to form and to reach their full sizes.

All the crystals were cryoprotected using their mother liquor supplemented with 15% glycerol and flash-frozen by quickly dipping into liquid nitrogen. The X-ray diffraction data were collected on beamline BL17U1 at Shanghai Synchrotron Radiation Facility (SSRF, Shanghai, China) at cryogenic temperature, maintained with cryogenic system. One single crystal was used for the data collection; data processing was carried out using the HKL2000 or HKL3000 programs (46). The data collection and processing statistics were summarized in Supplementary Table S1.

### Structure determination and refinement

The DNA1 structure was solved by the SAD (single anomalous diffraction) method (47) using the data collected at the peak wavelength (0.94967 Å) of Pb^2+^; the hkl2map program (48) was utilized during this process. Based on the electron density maps, the initial DNA1 model was manually built with the graphic program Coot (49). The higher resolution data collected at the wavelength of 0.97928 Å were utilized for the building and refinement of the final DNA1 model, which was obtained by molecular replacement method using the initial DNA1 model as the search model (50). The structure was refined with the Refmac5 program (51) embedded in the CCP4i suite. The refinement statistics were also summarized in Supplementary Table S1.

### Circular dichroism (CD) experiments

All the DNAs (Supplementary Table S2) utilized in the circular dichroism (CD) experiments were synthesized by solid phase synthesizer and purified by High Performance Liquid Chromatography (HPLC). All samples were prepared by dissolving the purified oligonucleotides in the crystallization buffer that contains 100 mM CAPS-NaOH pH 10.5, 200 mM Li_2_SO_4_ and 2.0 M (NH_4_)_2_SO_4_. All solutions were heated to 85°C for 3 min, then cooled slowly to room temperature and stored at 4°C for overnight. CD studies were carried out in utilizing a Jasco-815 CD spectrometer in a quartz cell with a 10-mm path length. CD spectra were collected from 350 to 200 nm and with a scanning speed of 100 nm/min. The bandwidth was set to 1.0 nm, and the digital integration time was 1.0 s. All CD spectra were baseline-corrected against the blank buffer.

### Nuclear magnetic resonance experiments

The DNAs utilized in NMR studies were synthesized using Dr Oligo 48 DNA synthesizer (Biolytic Lab Performance Inc, USA), dialyzed successively three times against ddH_2_O, lyophilized and dissolved in 500 μl of three differ buffers. Buffer 1 and 2 are composed of 50 mM CAPS-NaOH pH 10.5, 1.0 M (NH_4_)_2_SO_4_ and 100 mM Li_2_SO_4_, and 50 mM CAPS-NaOH pH 9.2, 100 mM (NH_4_)_2_SO_4_ and 100 mM Li_2_SO_4_, respectively. Buffer 3 is composed of 80 mM NaH_2_PO_4_/Na_3_PO_4_ pH 6.8, 100 mM (NH_4_)_2_SO_4_ and 100 mM Li_2_SO_4_. A total of 10% D_2_O is included in all NMR buffers. The concentrations of each NMR sample were typically about 1.5–2.0 mM. The one-dimensional ^1^H NMR spectra, with a spectral width 16 ppm and scanning number 2K, were performed at 20°C on a Varian Unity Inova 600 NMR spectrometer equipped with a triple resonances cryoprobe and pulsed field gradients.

### Gel electrophoresis

DNAs were dissolved in buffer 4 (40 mM Tris–HCl pH 8.0 and 200 mM KCl) with a final concentration of 20 μM. The samples were heated at 95°C for 5 min, then incubated immediately on the ice or cooled slowly to room temperature. A total of 2 μl DNA, 3 μl ddH_2_O and 5 μl native gel loading buffer were mixed, then 4 μl sample was applied to the native gel containing 8% acrylamide in Tris-borate-EDTA (TBE) buffer pH 8.3 supplemented with 50 mM KCl. The gel was run at 100 V for 50 min. Then, the gel was stained by Gelred and imaged by Gel-Imaging system.

## RESULTS

### Overall structure of DNA1

The DNA1 sequence was originally designed to mimic the substrate of Pb^2+^-dependent 8–17 DNAzyme as previously reported (52,53). Though 8–17 DNAzyme was present in the crystallization sample, only DNA1 sequence crystallized out under the conditions composed of 100 mM CAPS-NaOH pH 10.5, 200 mM Li_2_SO_4_ and 2.0 M (NH_4_)_2_SO_4_; the crystal diffracts to 1.45 Å resolution (Supplementary Table S1). The structure was solved by the SAD method using the anomalous signal of Pb^2+^, which was also present in the crystallization sample and bound to DNA in the structure; the structure was refined with the Refmac5 program, the final R-factor and *R*_free_ values are 13.7 and 16.9%, respectively. The crystal belongs to P2_1_2_1_2 space group, it contains two DNA1 molecules per asymmetric unit. Via the 2-fold symmetry along the long axis, DNA1 can assemble into quadruplex with four DNA strands parallelly orientated (Figure 1). DNA1 is 17 nt long in length; whereas, there are more than 300 ordered water molecules observed in the DNA1 quadruplex structure (Supplementary Figure S1). These water molecules are mainly located within the grooves and along the phosphate backbone of the quadruplexes; the extensive hydrogen bond (H-bond) interactions between the nucleotides and the water molecules may play important role in the folding and stabilization of the quadruplexes.

### Conformations of the homogenic G-, A-, C- and T-tetrads

**Figure 2.**
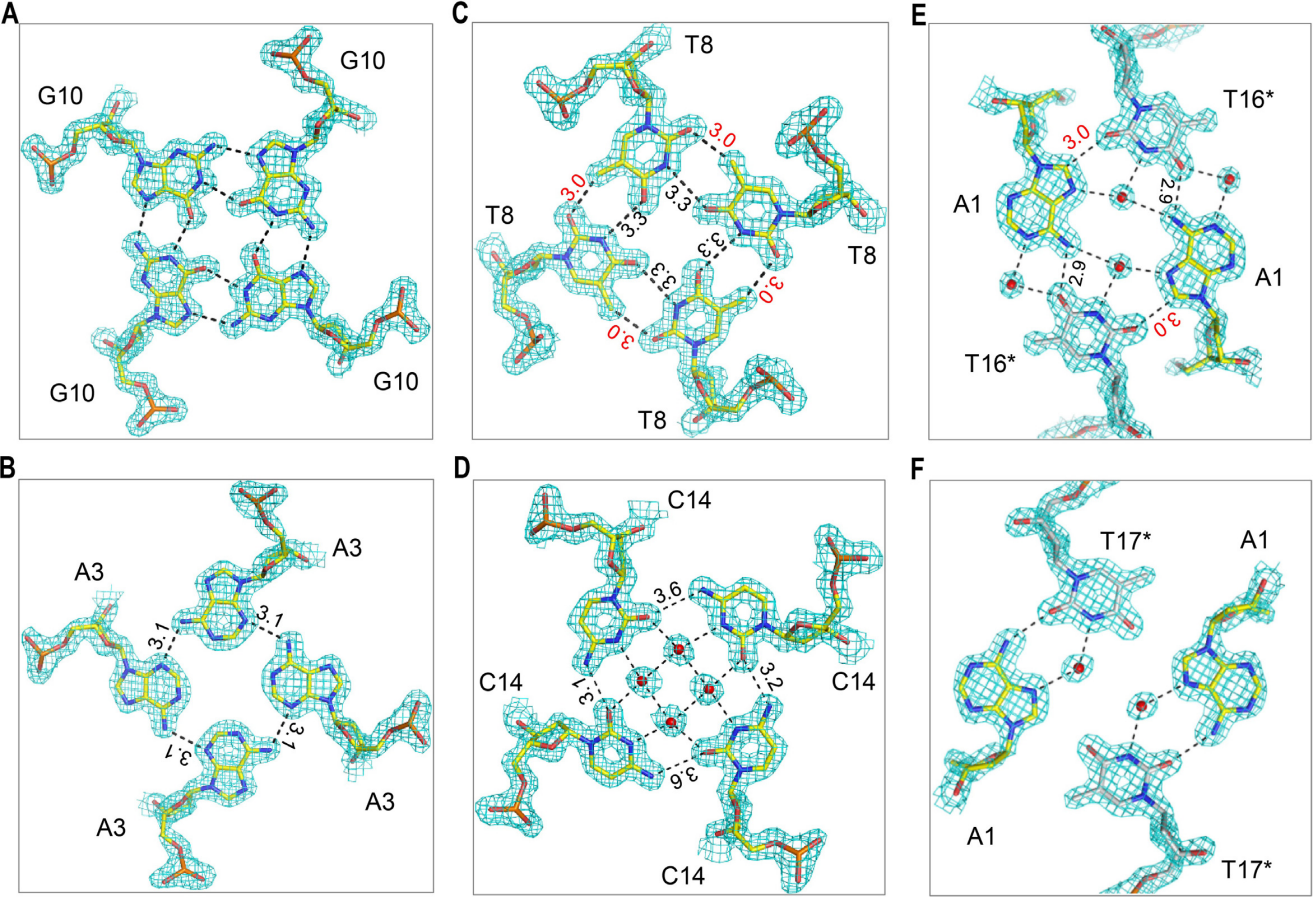
The representative tetrads observed in the DNA1 structure. The detailed conformations of (**A**) the G10-tetrad, (**B**) the A3-tetrad, (**C**) the T8-tetrad, (**D**) the C14-tetrad, (**E**) the A1:T16*:A1:T16* tetrad and (**F**) the A1:T17*:A1:T17* tetrad. The DNAs and the water molecules are shown as sticks and spheres, respectively. The 2F_o_-F_c_ electron density maps are contoured at 1.5 σ level. In panel (C) and (E), the unusual C-H⋯O hydrogen bonds stabilizing the T-tetrad and A:T:A:T tetrad are labeled by red numbers.

The central region (G2–G15) of DNA1 contains several non-G residues, most of which form tetrad structures. The middle G-tetrads are mainly stabilized by the regular Hoogsteen hydrogen bond (H-bond) interactions among the G residues (Figure 2A). Similar interactions have been observed in many reported quadruplex structures, including recent NMR structure of human telomere RNA ORN-1 (32). Several A-tetrads were also captured in the ORN-1 structure; like the structure of (^Br^dU)r(GAGGU) (54), the A-tetrads observed in ORN-1 all form H-bonds between their N7 and N6 atoms. In DNA1 structure, instead of the N7 atoms, the N3 atoms form H-bonds with the N6 atoms of the pairing adenines, resulting in a bigger central cavity in the A-tetrad (Figure 2B); similar A-tetrad has been observed in the rU(^Br^dG)r(AGGU) structure (36). Compared to the G-tetrads, the average distance between the C1′ atoms of the diagonal residues is ∼1.6 Å shorter in the N6-N3 H-bond stabilized A-tetrads. Though the residues all adopt anti-conformation, superimpose of the A- and G-tetrads (Supplementary Figure S2A) clearly shows that the adenine nucleobases are counter-clockwisely rotated about 20° with respect to guanines.

Both T8 and T12 form identical T-tetrads in the DNA1 structure; the detailed conformation of T8-tetrad is shown in Figure 2C. T-tetrads have been observed in one NMR quadruplex structure (PDB ID: 1EMQ) previously reported (31), in which the T-tetrads form stable H-bonds (2.8–3.0 Å) between their N3 and O4 atoms. However, as indicated by the long distance (3.3 Å), the pairing thymine residues only form relatively weak H-bonds between their N3 and O4 atoms in DNA1 quadruplex. Instead, they form unusual C-H⋯O hydrogens bond between their methyl carbons and O2 atoms. The distances of the C-H⋯O hydrogen bonds are all around 3.0 Å, suggesting that these interactions are very stable and may play critical role in the T-tetrad formation and stabilization. As revealed by structural superposition (Supplementary Figure S2B), the nucleobase and sugar pucker orientations of T and G are similar in the T-tetrad and G-tetrad. The average distance is 15.1 Å between the C1′ atoms of the diagonal T8 residues, whereas it is 16.3 Å between the C1′ atoms of the diagonal G10 residues.

The C-tetrad was formed by the C14 residues (Figure 2D). Like the A-tetrads, the nucleobases of the C-tetrad are also counter-clockwise rotated with respect to the nucleobases of the G-tetrad (Supplementary Figure S2C). Similar to the A- and T-tetrads, the averaged C1′-C1′ distance (13.8 Å) of the diagonal C14 residues is also significantly shorter than the G-tetrads. Structural analysis (Supplementary Figure S2D) reveals that the cytosine sugar puckers adopt two different conformations, C4′-exo (for strands A and C) or C1′-exo (for strands B and D). The C14 residues do not form stable interactions with each other, indicated by the long distances (3.2 Å or 3.6 Å) between the O2 and N4 atoms of the neighboring residues. Instead, this C-tetrad was mainly stabilized by the four highly conserved water molecules, which locate in the middle of tetrad plane with a rectangle arrangement and form stable H-bonds (2.8 Å) with the O2 and N3 of the four cytosine residues. The average distance between the neighboring water molecules is 2.9 Å, suggesting the stable formation of a hydrogen-bonding network with the C-tetrad plane.

### Various conformations of the A:T:A:T tetrad

The terminal A1 residues do not form homogeneous A-tetrads; instead, the four A1 residues of one quadruplex stack with each other within two layers and form tetrad planes with two of the T16*-T17* steps of symmetry-related quadruplex molecules (Supplementary Figure S3A). As depicted in Figure 2E, both A1 and T16* adopt *anti*-conformations in the A1:T16*:A1:T16* tetrad, which is stabilized by various types of interactions including direct H-bond interactions (2.9 Å, between the N6 atom of A1 and the O4 atom of T16*), and indirect water-mediated H-bond interactions. There are two highly conserved water molecules located at the center of the tetrad plane, with the averaged distance between each water molecule and its interacting N6 (of A1), N7 (of A1) and N3 (of T16*) atoms around 2.9 Å. A1 and T16* residues also interact with each other by forming an unusual C-H⋯O bond between their C8 and O2 atoms. As indicated by the short distances (3.0 Å), these C-H⋯O bonds might be strong and further contribute to the formation of this A:T:A:T tetrad.

A1 residues adopt *syn*-conformation in the A1:T17*:A1:T17* tetrad (Figures 1C and 2F), which is different from the one in the A1:T16*:A1:T16* tetrad. In addition, it is also noteworthy that the sugar pucker conformation of the A1 residues is also different in the two tetrads, adopting C2′-endo and C2′-exo pucker respectively in A1:T16*:A1:T16* and A1:T17*:A1:T17* tetrads (Supplementary Figure S3B). Like the A1:T16*:A1:T16* tetrad, the A1:T17*:A1:T17* tetrad is also stabilized by direct H-bonds and water-mediated H-bonds between A1 and T17* bases; however, the detailed interactions are different in the two A:T:A:T tetrads. The N6 atom of A1 forms one H-bond (2.9 Å) with the O2 atom of T17* instead of the O4 atom. The two water molecules locating in the middle of the tetrad plane form H-bonds with the N3 (of T17*) and N7 (of A1) atoms with the average O-N distance of 2.7 Å. As indicated by the closest distance (3.4 Å) between them, the two T-A pairs do not form strong interaction with each other in the A1:T17*:A1:T17* tetrad structure. Considering that all the previously reported A:T:A:T tetrads, which are observed in human telomere RNA (55) and DNA (6) quadruplex structures, are mainly stabilized by the Watson-Crick A:T pairing and one additional H-bond between the N6 and O4 atoms of the A:T pairs, our structure provides additional insights into the conformational diversity and complexity of these heterogenetic A:T:A:T tetrads.

### Kinking and stabilization of A7

**Figure 3.**
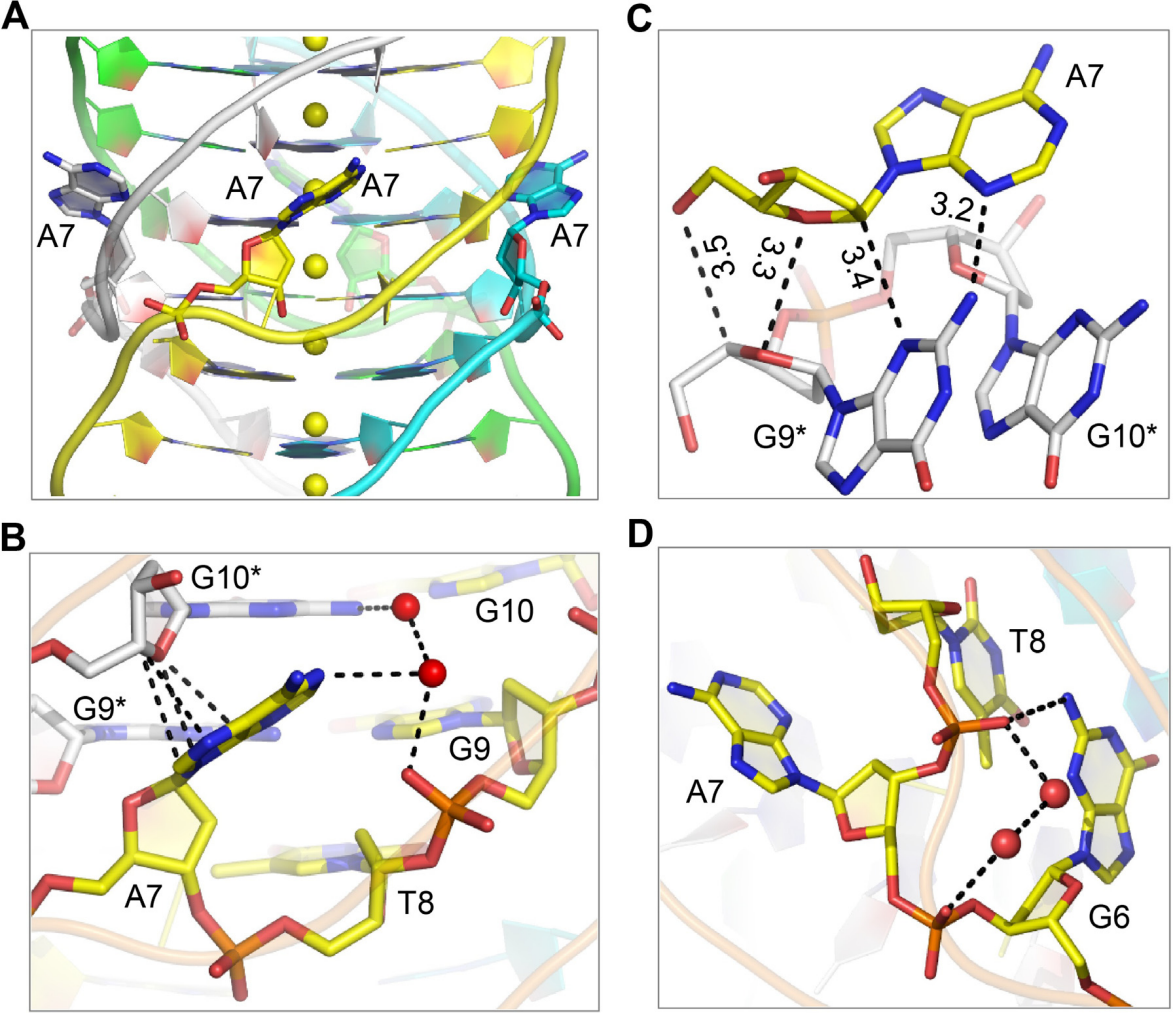
Kinking and stabilization of A7 residues. (**A**) Close-view showing the kinking of A7 residues, which are shown as sticks. (**B**) Stacking interaction between the nucleobase of A7 and the sugar pucker of G10* of the neighboring strand. (**C**) Shape complementation between A7 and the neighboring G9* residues. (**D**) Tilting of the T8 phosphate backbone and its interaction with G6. The DNAs and the water molecules are shown as sticks and spheres, respectively.

It is known that A-tetrad is the second most frequently observed tetrad structure besides G-tetrad. However, even though all the thymine and cytosine residues in DNA1 form tetrads, the middle A7 residues form a kink in our structure (Figure 3A). Overall, the kinked A7 residue is stabilized by several different interactions. As shown in Figure 3B, the adenosine imidazole ring of A7 packs tightly with the G10* residue of the neighboring strand, forming extensive hydrophobic interactions between the ring atoms (N7, C8, N9 and C4) of A7 and the sugar pucker atoms (C4′ and O4′) of G10* with the distances ranging from 3.2–3.4 Å; similar hydrophobic interactions have been previously proposed (56). Via water-mediated H-bonding, the N6 atom of A7 interacts with the OP2 atom of G9 and the N2 atom of G10*. A7 fits in the helical groove of the quadruplex and is close to G9* (Figure 3C), suggesting they may form weak hydrophobic interactions; the distance is 3.5 Å between the O5′ atom of A7 and the C4′ atom of G9* and is 3.3 Å and 3.4 Å between the O4′ atoms of both A7 and G9* and between the C1′ atom of A7 and the N3 atom of G9*, respectively. Besides these close contacts, one weak H-bond interaction (3.2 Å) formed between the N3 atom of A7 and the N2 atom of G9* may also play a role in orienting the kinking of A7.

Formation of the kink only has very little impact on the orientation of the backbone phosphate of A7; similar to many other residues in the structure, the phosphate group of A7 is exposed toward the outside of the groove. T8 adopts a distorted conformation (Figure 3D). Compared to A7 and all the other surrounding residues, the phosphate group of T8 is tilted more than 90° toward the groove and its OP2 atom forms a strong H-bond (2.9 Å) with the N2 atom of G6. Via two water molecules, the OP2 atom of T8 forms additional H-bond with the OP2 atom of A7, which may further stabilize the conformation of A7.

In terms of the geometric effects of this unique A7 kink to the neighboring residues, the distance between the P atoms of G6 (of strand A) and G4* (of strand B) is 8.9 Å (Supplementary Figure S4), which is identical to the one between the P atoms of G12 (of strand C) and G10* (of strand D); similar P-P distances were also observed in other regions of DNA1 and the structures of many reported quadruplexes, indicating that kinking of A7 does not affect the conformation of the whole structure. However, kinking of A7 pushes the backbones of G6 and A7 and the backbones of T8* and G9* of two neighboring strands toward each other, resulting in a tightly compacted groove, which is only about 4.1 Å wide; meanwhile, two other grooves (one between A7 and A5* of two neighboring strands and one between G9* and G11 of another two neighboring strands) are widened for 1.5 Å and 2.5 Å, respectively.

### Central cation binding

**Figure 4.**
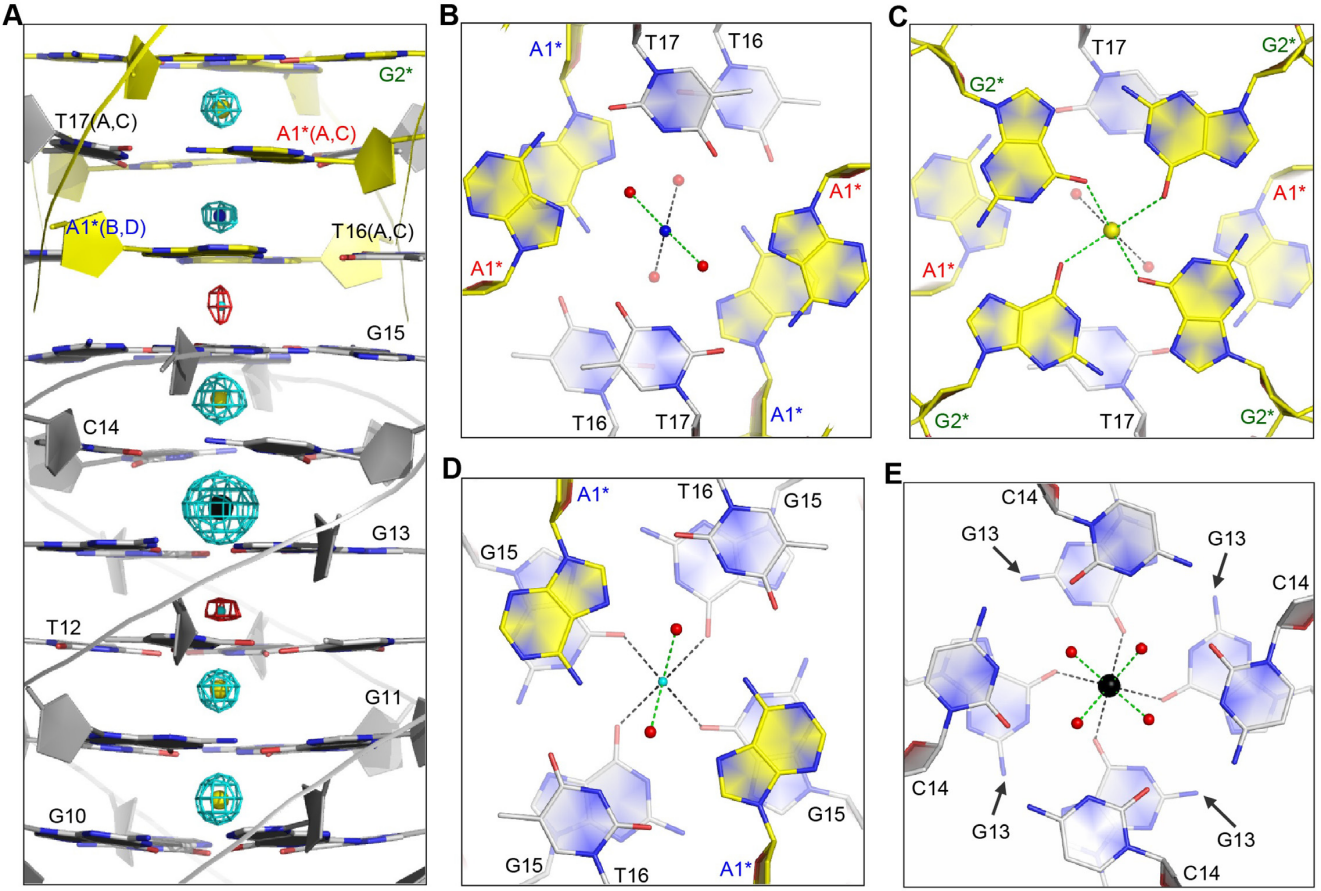
Binding of the central ions. (**A**) Arrangement of the ions in the central channel of the DNA1 structure. The Li^+^, NH_4_^+^, Na^+^ and Pb^2+^ ions are shown as spheres in cyan, blue, yellow and black, respectively. The 2F_o_-F_c_ electron density maps of Li^+^ ions are contoured at 0.5 σ level, whereas it is contoured at 1.5 σ level for all other ions. (**B**) Coordination of the NH_4_^+^ ion bound in-between the A1*:T16:A1*:T16 and A1*:T17:A1*:T17 tetrads. (**C**) Coordination of the Na^+^ ion bound in-between the A1*:T17:A1*:T17 tetrad and the G2*-tetrads. (**D**) Coordination of the Li^+^ ion bound in-between the A1*:T16:A1*:T16 tetrad and the G15-tetrad. (**E**) Coordination of the Pb^2+^ ion bound in-between the G13-tetrad and the C14-tetrad. The coordinating water molecules are shown as red spheres in panels (B–E). Nucleotides from the symmetry-related quadruplex are indicated by asterisks.

Besides tetrad stacking, the cation-tetrad interaction is another essential driving force in the folding and stabilization of DNA1 quadruplex structure. In addition to Na^+^, there are three more types of cations existing in the crystallization sample (Pb^2+^) or buffer (Li^+^ and NH_4_^+^). In the DNA1 structure, one cation has been captured and sandwiched between each neighboring tetrad planes (Figure 4A).

As one of the most common cofactors of G4, Na^+^ ion accounts for the majority cations bound in the DNA1 structure. Beside the common G-tetrad layers, Na^+^ ions are also found in-between various other tetrad layers (Supplementary Figure S5). When bound in-between the G- and A-tetrads (or A- and G-tetrads), Na^+^ ion only coordinates with the O6 atoms of G-tetrad, forming pyramid-like four-coordinated conformation. Na^+^ ion does not interact with the A-tetrad, but two Na^+^ ions separated by the A-tetrad can form strong metallophilic attraction, indicated by the short distance (∼2.9 Å) between them. The metallophilic attraction has been observed in various nucleic acid structures (57–59), it may also play important roles in the folding and stabilization of DNA1 structure. When bound in-between the G- and T-tetrads (or T- and G-tetrads), Na^+^ ion coordinates with both of the O6 atoms of G-tetrad and O4 atoms of T-tetrad, forming eight-coordinated conformation, which is similar to the one bound in-between the common G- and G-tetrads.

One cation (Figure 4B) is bound in-between the two A:T:A:T tetrads. Instead of direct coordination with the A or T residues, this cation forms H-bonds with four water molecules, which arrange like a pyramidic cone and stabilize the A:T:A:T tetrads. The pyramidic-like geometry suggests that the bound cation is NH_4_^+^; similar four-coordinated NH_4_^+^ ion has also been observed in several other high-resolution nucleic acid duplex structures (60–62). Locating in the center of the pyramidic cone, the distance between the NH_4_^+^ ion and the four water molecules are all around 2.7 Å. The A:T:A:T tetrads are flanked by two G-tetrads, composed of G2* and G15 residues, respectively. Interestingly, one Na^+^ ion is bound in-between the A1*:T17:A1*:T17 and G2* tetrads (Figure 4C). The very weak electron density suggests that it is one Li^+^ ion bound in-between the G15 and A1*:T16:A1*:T16 tetrads (Figure 4D). Both of the Na^+^ and Li^+^ ions are six-coordinated; in addition to the four O6 atoms of G-tetrad, they also coordinate with the two water molecules stabilizing the A:T:A:T tetrads.

Pb^2+^ ions have been known to have strong binding affinity to some nucleic acid sequences, such as the thrombin-binding aptamer (TBA) (41), and stabilize or switch the structures of many quadruplexes. One well-defined Pb^2+^ ion is captured in our DNA1 structure, representing the first quadruplex–Pb^2+^ complex structure. Besides the anomalous signal that we used to solve the overall DNA1 structure, the identity of the Pb^2+^ ion is further supported by its strong electron density (Supplementary Figure S6). The Pb^2+^ is bound in-between the G13-tetrad and the C14-tetrad (Figure 4E) with eight coordination form. In addition to the four O6 atoms of G13-tetrad, the Pb^2+^ ion also coordinates with the four water molecules that stabilize the C14-tetrads. The average coordinating distance between the Pb^2+^ ion and the O6 atoms of G13 residues is 2.8 Å; and, it is only 2.5 Å between the Pb^2+^ ion and the water molecules. Compared to the average Na^+^-coordinating distance (3.0 Å), the Pb^2+^-coordinating distance is significantly shorter, suggesting that the Pb^2+^-coordination could be more stable than the Na^+^-coordination. It is worth notice that Pb^2+^-coordination of DNA-1 is different from TBA, which was predicted to coordinate Pb^2+^ using the central G-tetrad core, suggesting that quadruplex might be flexible in coordinating Pb^2+^. It is also worth notice that, instead of Pb^2+^, one Na^+^ ion is bound in-between the C14-tetrad and the G15-tetrad (Supplementary Figure S6), indicating that the Pb^2+^-coordination might be tetrad orientation-dependent.

### Characterization of residues important for DNA1 quadruplex formation

It has been known that the G4 structures are sensitive to the base components of the sequence, and even small modifications could cause dramatic structural changes (63–65). To investigate the sequence dependence of this novel DNA quadruplex, we did systematic sequence mutation of DNA1 (Supplementary Table S2) and carried out the CD spectrum analysis, particularly targeting the terminal residues, the kinked A7 and the A7-interacting nucleotides.

**Figure 5.**
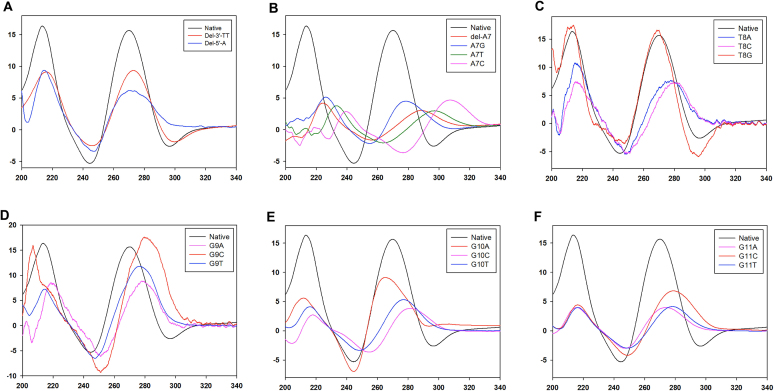
CD analysis of the native and mutated DNA1 sequences. (**A**–**F**) Comparison of the native DNA1 and the terminal truncated, the A7 deleted or mutated, the T8 mutated, the G9 mutated, the G10 mutated and the G11 mutated sequences, respectively. The *x*-axis is wavelength (nm) and the *y*-axis is CD absorption Δϵ (M^−1^cm^−1^).

Depend on their topologies, G4 exhibit characteristic CD spectra (66). Parallel quadruplexes normally exhibit a maximum positive signal around 260 nm and a negative signal around 240 nm. Whereas, the antiparallel quadruplexes show a characteristic positive signal around 295 nm and a negative signal around 260 nm. The CD spectrum of native DNA1 gives a positive peak at 269 nm, a negative peak at 244 nm and an additional positive peak at 210 nm, which are very similar to other parallel G4. Deletion of 3′-T16T17 or 5′-A1 residues retains the absorption patterns but decreases the overall CD signals (Figure 5A), indicating that the three terminal residues are not directly involved in the quadruplex formation but may affect the molecular packing, which is consistent with our structural analysis. Considering A7 is the only residue forming unique kink in the overall DNA1 structure, we next investigated the consequence of this residue. Interestingly, deletion or mutation of A7 to any other nucleotides all causes dramatic peak shifting and decreasing of the positive 269 nm peak, as well as the disappearance of negative 240 nm (Figure 5B). Particularly, when A7 was mutated to C, the overall CD spectrum showed the formation of an anti-parallel like quadruplex structure (the pink curve in Figure 5B), which suggests that A7 is critical for the quadruplex formation, most likely due to its kinking and extensive interactions with the surrounding residues as shown in Figure 3.

T8 is the only residue projecting its phosphate group into the groove. Replacing T8 with either A8 or C8 affects the formation of the overall structure (Figure 5C); however, as indicated by the almost identical CD spectra (the red curve in Figure 5C), replacing T8 with G8 does not have any structural perturbation on the quadruplex formation, probably due to the similar nucleobase orientations of T-tetrad and G-tetrad (as shown in Supplementary Figure S2B). Among the three consecutive G residues G9–G11, G9 has relative small impact on the quadruplex formation, as indicated by the small CD signal change (Figure 5D). In contrast, replacing G10 and G11 by other nucleotides (A, T or C) all lead to clear shifting and/or decreasing of the signals (Figure 5E and F), indicating that both G10 and G11 residues are important in the folding or stabilization of the quadruplex.

Deletion of A7 results in a G6T8G9 motif, which is the same fragment as G11T12G13 in the native DNA1 sequence. The context upstream of G6 is G4A5; mutation of A5 could form another GTG (G4T5G6) motif. As depicted in Supplementary Figure S7, the overall effects of A5 are very similar to the G10 residue, with either shifted or decreased CD absorptions. These observations indicate that GTG motif might be a factor disrupting the DNA1 quadruplex formation and A5 is an important sequence context in folding of the structure.

### Molecular packing of DNA1

**Figure 6.**
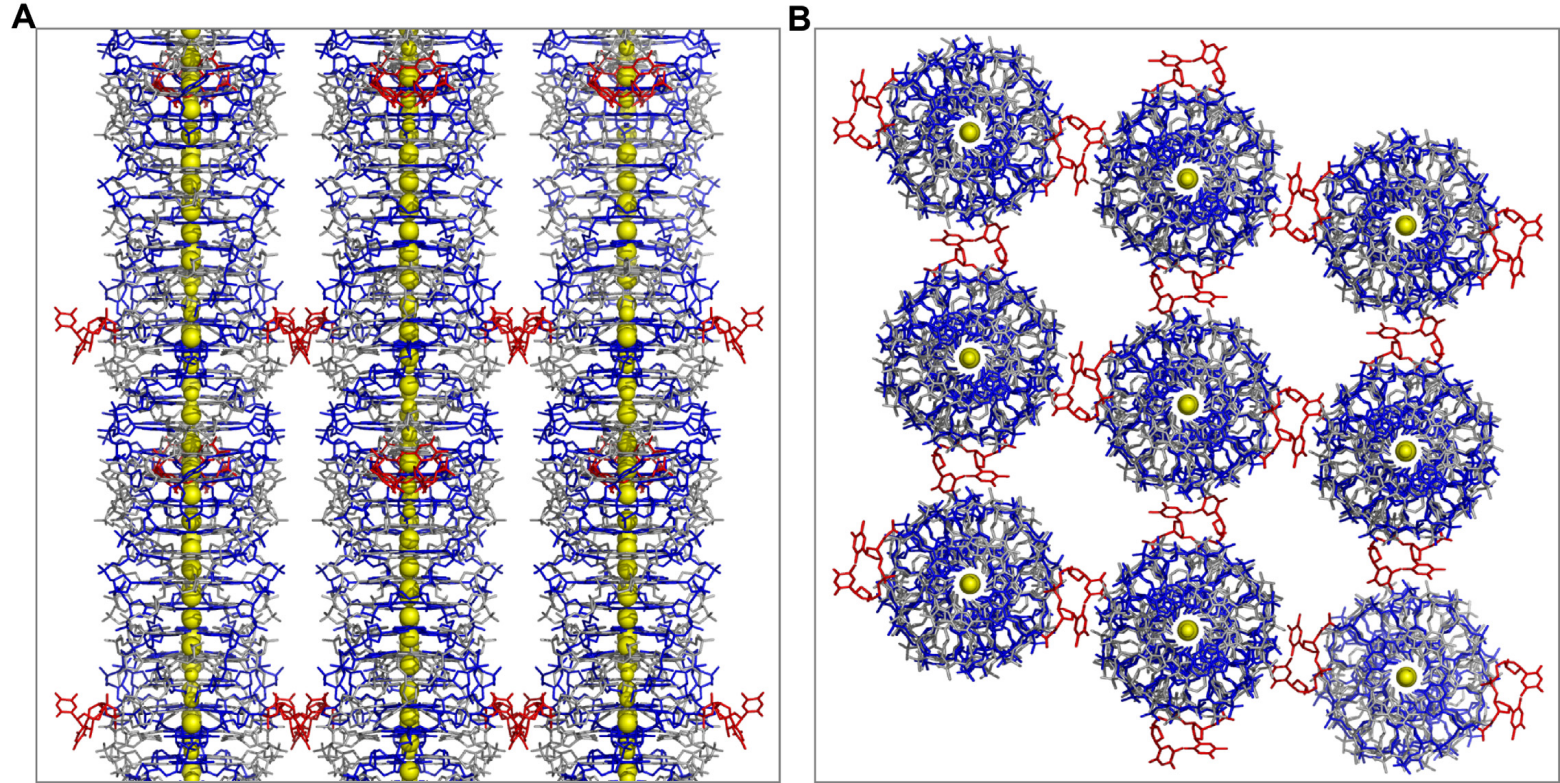
Molecular packing and nanowire structure formation of DNA1 quadruplex. (**A**) A view perpendicular to the helical axis showing the nanowire-like structure formed by multiple DNA1 quadruplexes. (**B**) A view along the helical axis showing the loose interaction between the individual nanowire structure. The DNAs are shown as sticks in gray or blue; the T16 and T17 residues involved in the nanowire packing are colored in red. All the cations bound at the central channels of the nanowires are shown as yellow spheres.

Via the terminal A:T:A:T tetrad formation (as shown in Figure 2E and F), the symmetry-related DNA1 quadruplexes form head-to-tail stacking in the crystal lattice (Supplementary Figure S3A). As a result, multiple DNA1 quadruplexes assemble into an infinite nanowire-like structure, which captures one cation in-between each tetrad layer (Figure 6A). Among the four T16–T17 steps of the DNA1 quadruplex, two of them are directly involved in the A-T tetrad formation; whereas the other two are flipped out of the quadruplex core and interact with the neighboring residues in the next DNA1 molecule, further expanding the nanowire structure (Figure 6B). The nucleobases of the flipped out T16 and T17 are approximately perpendicular to each other. Via their N3 atoms, T16 and T17 form two H-bonds with one symmetry-related quadruplex: one with the OP2 atom of G9 and one with the OP1 atom of G11 (Supplementary Figure S3C). The O2 atom of T16 also forms one H-bond with the N6 atom of A7 of the neighboring quadruplex molecule, which further stabilizes the conformations of these two T restudies. As revealed by the identical conformations of all the A7 residues, the T16 H-bond interaction does not affect the kinking of A7. The interactions between the individual quadruplex nanowires are not very strong; besides the H-bonds mediated by T16 and T17, no other direct interactions between the nanowires are identified in the crystal lattice.

### Solution studies of DNA1

**Figure 7.**
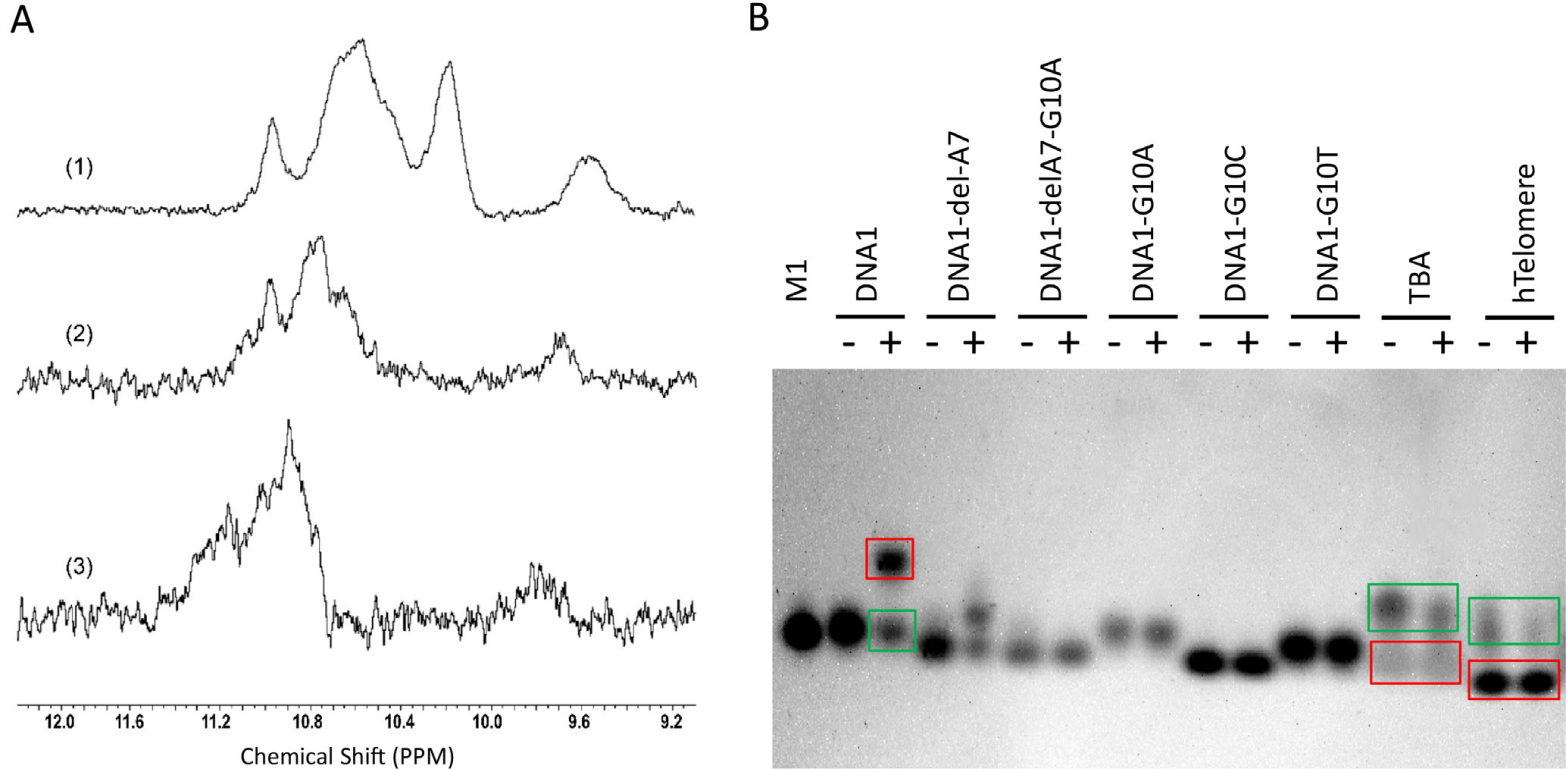
Solution studies of DNA1. (**A**) Overlay of the imino regions of 1H NMR spectra of DNA1 dissolved in buffers 1, 2 and 3. (**B**) Native gel analysis of DNA1, DNA1 mutants, TBA and hTelomere dissolved in buffer 4. The single-stranded and quadruplex structures of DNA1, TBA or hTelomere are highlighted with green and red boxes, respectively. The denatured and annealed samples are indicated by ‘–’ and ‘+’ labels, respectively. M1 is one single-stranded DNA markers with length of 17 nt (sequence in Supplementary Table S2). The detailed compositions of buffers 1–4 are given in the ‘Materials and Methods’ section.

Though DNA1 can assemble into quadruplex in the crystal structure; the crystals were grown under the condition with very basic pH (10.5) and high salt concentration (200 mM Li_2_SO_4_, and 2.0 M (NH_4_)_2_SO_4_). To test whether DNA1 can form quadruplex under other conditions, we performed NMR studies. As depicted in Figure 7A, the imino proton spectrum of DNA1 showed clear peaks between 9.2 and 11.6 ppm, which are characteristic peaks of quadruplexes in solution, under all the three tested buffers. The pH values are 10.5, 9.2 and 6.8 for buffer 1, 2 and 3, respectively. Though high concentration (NH_4_)_2_SO_4_ is contained in buffer 1, buffer 2 and 3 only contain 100 mM Li_2_SO_4_ and 100 mM (NH_4_)_2_SO_4_. These observations indicated that DNA1 can form quadruplex in buffer with wide range of pH values and lower salt concentration.

To further support the quadruplex formation of DNA1, we also carried out native gel analysis. As depicted in Figure 7B, DNA1 can form quadruplex in buffer 4 (40 mM Tris–HCl pH 8.0, 200 mM KCl). Besides the quadruplex bands, the band corresponding to the single-stranded DNA1 were also observed on the gel, suggesting that the DNA1 quadruplex assembly might be very dynamic. DNA1 forms nanowire-like structure in the crystal; however, such structure was not observed on the native gel, which may due to the dynamic assembly of the quadruplex and formation of the terminal A:T:A:T tetrads. In consistent with previous studies, our gel analysis showed that TBA and human telomere (hTelomere) sequence can form intramolecular quadruplexes, which move faster than their corresponding single-stranded sequences (Figure 7B and Supplementary Figure S8). In contrast to TBA and hTelomere, the mobility of DNA1 quadruplex is significantly slower than the single-stranded DNA1 on the native gel.

Besides DNA1, we also analyzed several DNA1 mutants by native gel analysis. As depicted in Figure 7B, denaturation or annealing have no impact on the mobility of DNA1-G10A, DNA1-G10C or DNA1-G10T, suggesting that these four mutants mainly exist as single strand in solution. Interestingly, besides the single-stranded band, one slightly slower moving band was also observed on the gel for the annealed DNA1-delA7 mutant. DNA1-delA7 did not show typical absorption peak of quadruplex on the CD spectrum (Figure 5B), whether it can form other type of structure needs to be further investigated. In addition to DNAs with either deletion or mutation of single residue, we also synthesized the DNA1-delA7/G10A mutate with both A7 deletion and G10A mutation. DNA1-delA7/G10A contains two repetitive pattern GAGTG (G4A5G6T8G9 and G9A10G11T12G13) motifs with one overlapping G residue (G9). Like the three G10 mutants of DNA1, DNA1-delA7/G10A mainly exist as single strand (Figure 7B), suggesting that simple GAGTG repeats are not sufficient to maintain the quadruplex formation.

## DISCUSSION

In summary, we solved a high-resolution crystal structure of DNA1 (5′- AGAGAGATGGGTGCGTT-3′). Though it contains only one GGG motif, DNA1 can form parallel quadruplex. The overall structure of DNA1 quadruplex is similar to the dimeric B-raf quadruplex (67), which has a pseudo 2-fold symmetry along the long axis. In addition to the regular G-tetrad, the DNA1 structure also captures the homogenic A-, C- and T-tetrads, making it the first DNA quadruplex structure containing all the possible homogenic tetrads identified to date. The DNA1 structure also represents the first quadruplex–Pb^2+^ complex available; besides Pb^2+^, it also reveals the potential basis for Li^+^, NH_4_^+^ and Na^+^ binding within the base tetrads. Compared to other cations, we find that the binding of Na^+^ ion is much more flexible; in addition to the common G-tetrads, Na^+^ ion can also bound in-between the G-tetrad and other tetrads including A- and T-tetrad, contributing to its strong stabilization effect in quadruplex. Beside the grooves, several water molecules were also observed between the tetrads; via interacting with the cations, these water molecules may play a role in the quadruplex assembly. Through kinking or backbone phosphate group tilting, A7 and T8 adopt conformations that are very different from the neighboring G9, G10 and G11 residues. As revealed by the CD spectra, all the five residues are important for the DNA1 quadruplex formation.

The central region (G2–G15) of DNA1 forms 13 homogenetic tetrads stacking on each other, which are the longest continuous tetrads observed within single quadruplex structure. Via the heterogenetic A:T:A:T tetrad formation by the terminal A and T residues, multiple DNA1 quadruplexes assemble into long nanowire-like structure. Very recently, a nanowire-like metallo-DNA system has been reported with a high-resolution crystal structure (68). It consists of a short dodecamer DNA duplexes stabilized by silver-mediated base pairs, which cages a cluster of silver ions along the DNA helical axis and assembles together through interstranded overhanging G pairing and bulge A stacking. DNA1 quadruplex and the nanowire-like metallo-DNA share clear similarity in their structure and metal binding pattern. Unfortunately, due to the dynamic of the quadruplex, no nanowire-like DNA1 structure was found on the native gel. Currently, we are doing extensive screening, aiming to find DNA1-based sequences which are capable of forming longer quadruplex and could be used in DNA nanodevice development.

Our site-specific mutagenesis and biophysical studies showed that the central ATGGG motif of DNA1 plays important role in the quadruplex formation. ATGGG motif is present in many human genes and it has been predicted as a potential transcription-factor binding motif that interacts with transcription factors in response to interleukin I in human chondrocytes (69). Via searching through the public genome sequences, we found that the whole DNA1 sequence is present in the genomes of many species, such as *Scophthalmus maximus* chromosome 11 (5451668-5451684), *Ictalurus punctatus* (mRNA of protein phosphatase, 336–352), *Ovis canadensis canadensis* isolate 43U chromosome 7 (87187197-87187213), *Cyprinus carpio* genome assembly common carp genome (50610–59626). As revealed by the CD analysis (Figure 5A), deletion of A1 or T16T17 of DNA1 has no strong impact on the folding of DNA1 quadruplex. Compared to DNA1, the central GAGAGATGGGTGCG motif is present in much more species, such as *Triticum aestivum* chromosome 3D (609961015–609961028, 17378772–17378759, 1760924–1760937, 291964026–291964039), *Homo sapiens* chromosome 11q (clone: RP11-317J19;97229-97242), *Mus musculus* chromosome 5 (clone: RP23-393A24;135158-135171) and various HIV-1 isolates from China, Brazil, Russia and France. The 11th human chromosome is one of the most gene-rich and disease-rich chromosomes in the human genome (70,71). Though it needs to be further investigated, our studies revealed a novel DNA motif that could serve as a potential target in controlling the expression or translation of the genes related to human diseases.

## DATA AVAILABILITY

The authors declare no competing financial interests. The atomic coordinate and structural factors of DNA1 have been deposited in the Protein Data Bank (PDB, www.pdb.org) under the access code 6A85.

## Supplementary Material

Supplementary Data
