# Supplementary material for: High-resolution DNA quadruplex structure containing all the A-, G-, C-, T-tetrads
Source: Nucleic Acids Res. 2018 Oct 4;46(21):11627–38. doi: 10.1093/nar/gky902 (PMC6265469; doi:10.1093/nar/gky902)
Supplement: Supplementary Data [file gky902_supplemental_files.pdf]

Supplementary Data

for

High-resolution DNA quadruplex structure containing all the A-, G-, C-, T-tetrads

Hehua Liu[1,2], Rui Wang[3], Xiang Yu[1,2], Fusheng Shen[3], Wenxian Lan[4], Phensinee Haruehanroengra[3], Qingqing Yao[2], Jing Zhang[1], Yiqing Chen[1], Suhua Li[1], Baixing Wu[2], Lina Zheng[2], Jinbiao Ma[2], Jinzhong Lin[2], Chunyang Cao[4,*], JiXi Li[1,*], Jia Sheng[3,*], Jianhua Gan[1,*]

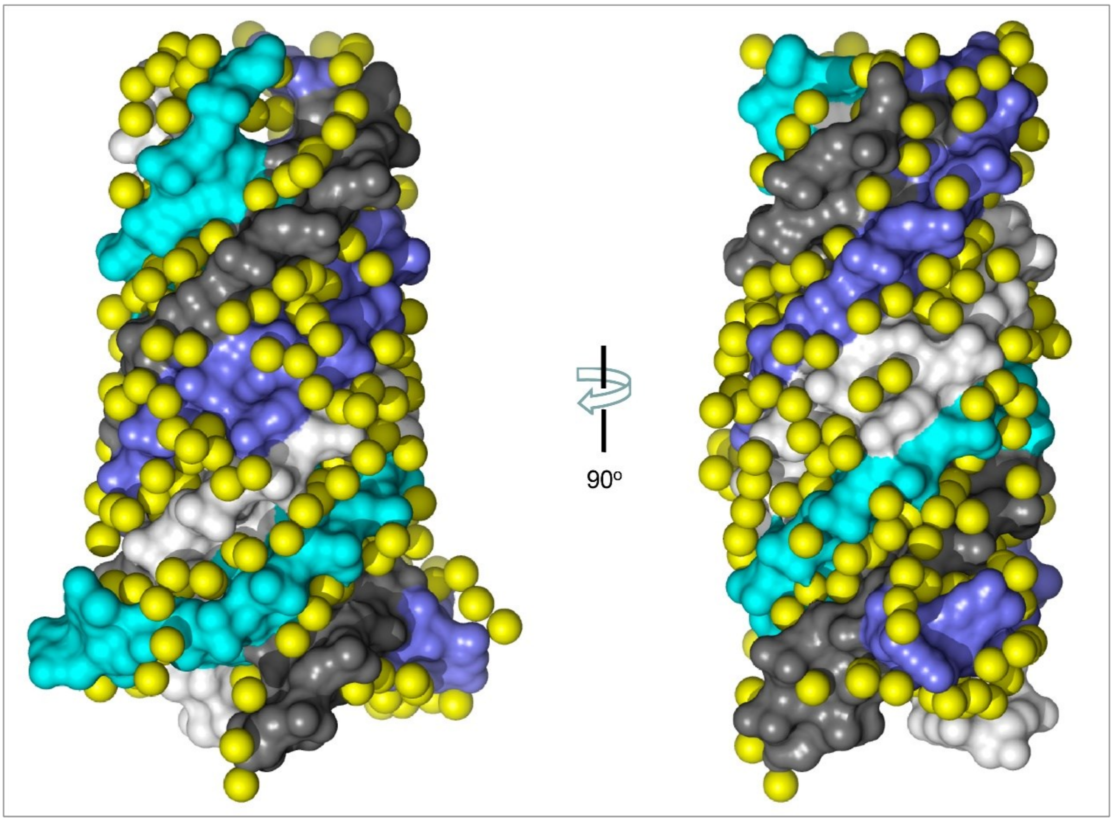

Supplementary Figure S1. Surface presentation of the DNA1 quadruplex structure. The four DNA strands are colored in gray, blue, white, and cyan, respectively. The water molecules are shown as yellow spheres.

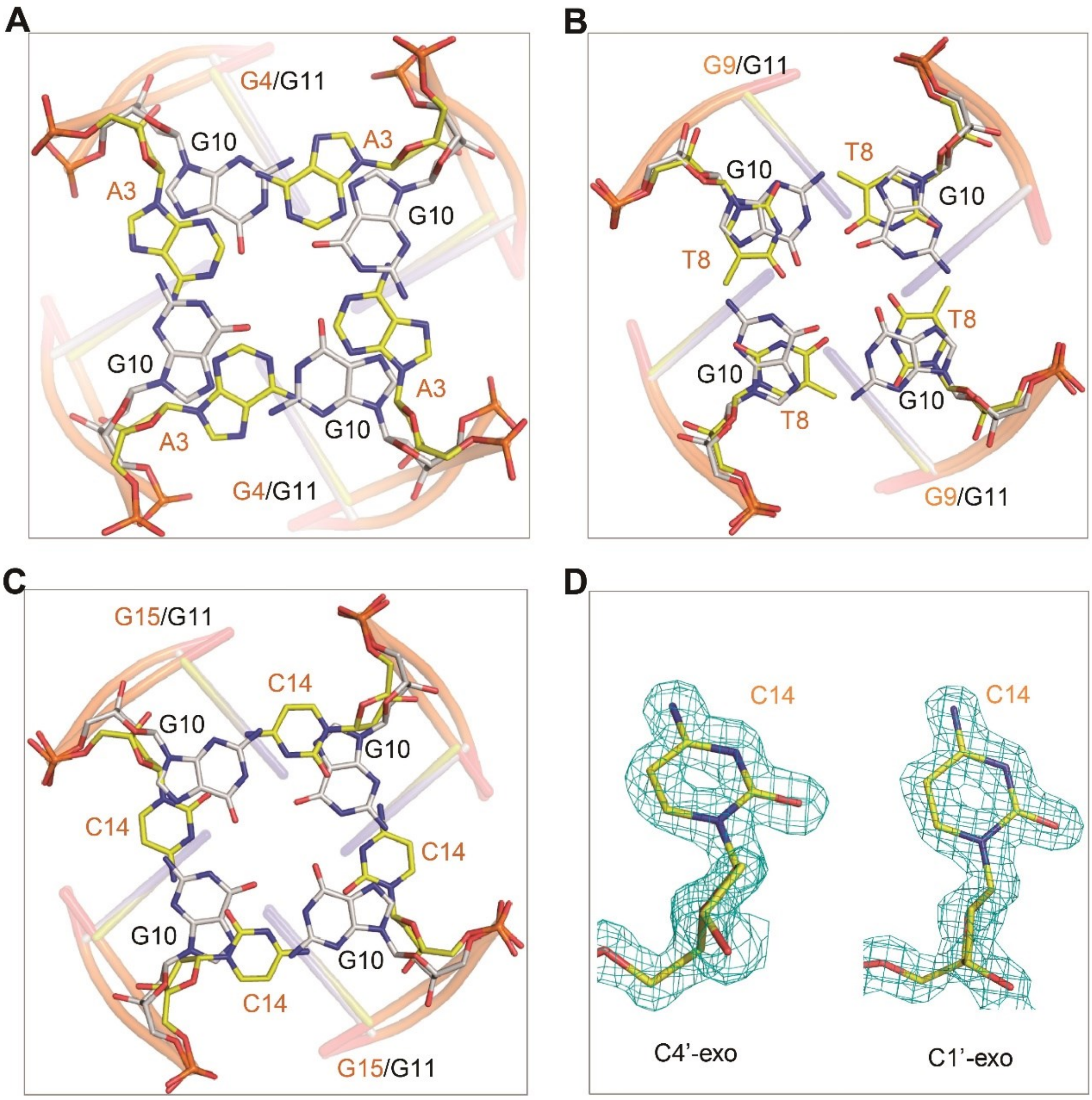

Supplementary Figure S2. Conformational comparison of the non-G-tetrad and the common G-tetrad. (**A**) Superposition of the A3/G4 and the G10/G11 steps showing the structural difference between the A-tetrad and G-tetrad. (**B**) Superposition of the T8/G9 and the G10/G11 steps showing the structural difference between T-tetrad and G-tetrad. (**C**) Superposition of the C14/G15 and the G10/G11 steps showing the structural difference between the C-tetrad and G-tetrad. (**D**) Stick view showing the different sugar pucker conformations adopted by the C14 residues. The $2F_o$-F_c electron density maps are contoured at 1.5 σ level.

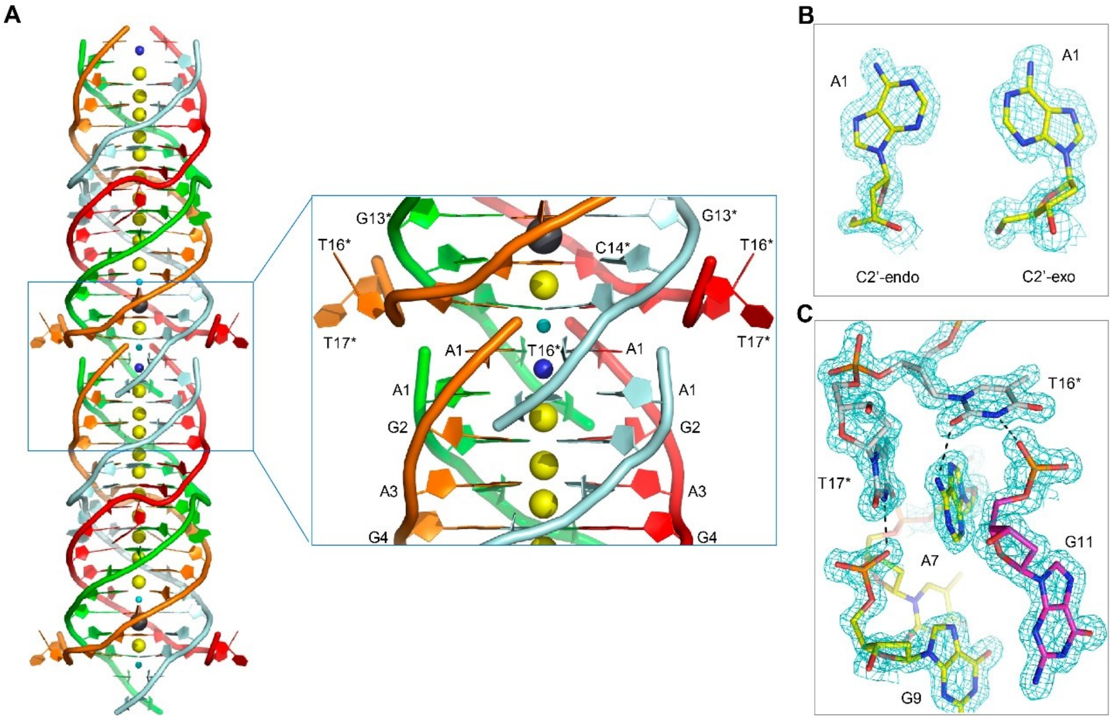

Supplementary Figure S3. Interactions mediated by the terminal adenine and thymine residues. (**A**) The A-T tetrads mediated head-to-tail packing of DNA1 quadruplexes. DNAs are shown as cartoon-and-ring mode with the four strands colored in cyan, red, green, and orange, respectively. The Li^+, NH_4^+, Na^+, and Pb^{2+} ions are shown as spheres in cyan, blue, yellow, and black, respectively. (**B**) The C2'-endo and C2'-exo conformations adopted by terminal A1 residues. (**C**) Interactions between the flipped out thymine residues (T16 and T17) and the neighboring quadruplex. The $2F_o$-F_c electron density maps are contoured at 1.5 σ level.

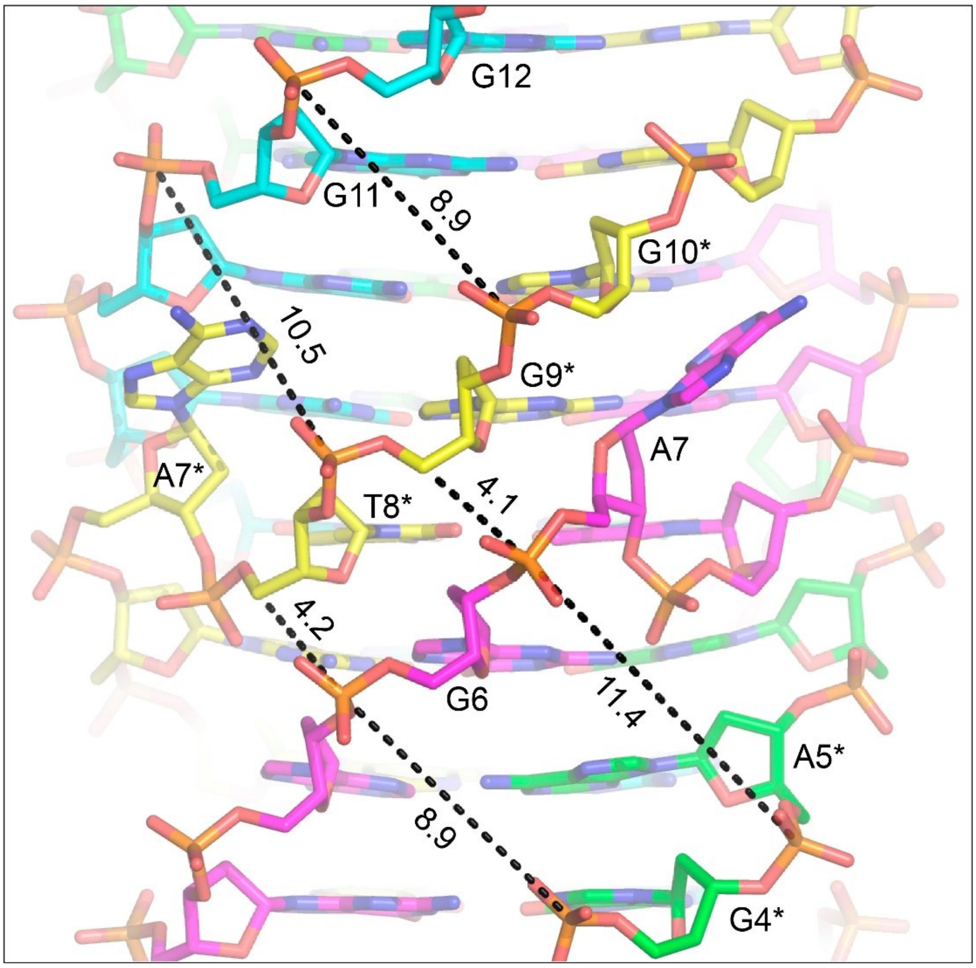

Supplementary Figure S4. A close view showing the A7 kink and the groove width between the neighboring strands. The DNAs are shown as sticks with the C-atoms colored in magenta, green, cyan, and yellow for strand A-D, respectively.

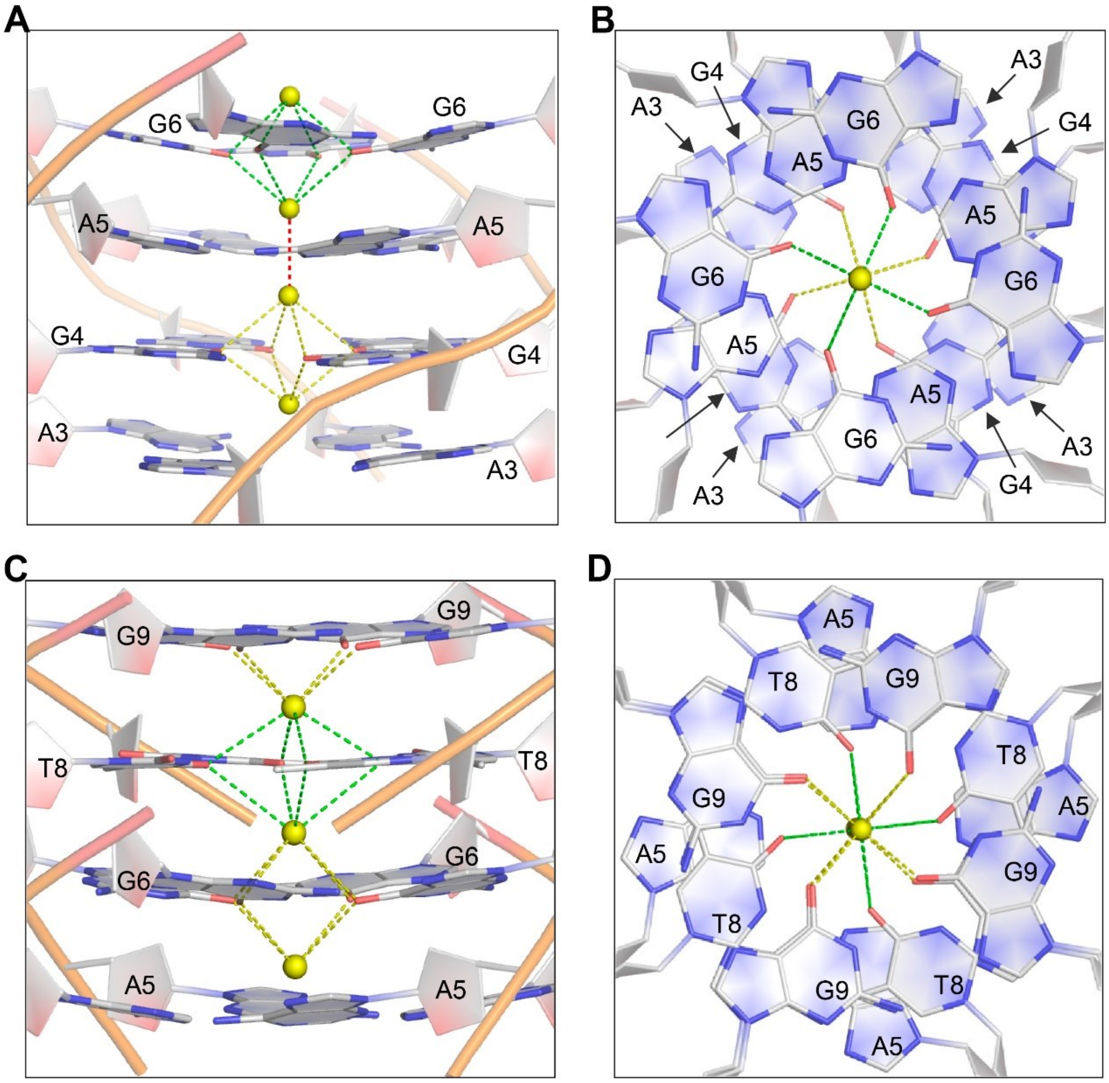

Supplementary Figure S5. Coordination of the central Na^+ ions in the DNA1 structure. (**A**) and (**B**) Coordination of the Na^+ ions in-between the G- and A- tetrads (or A- and G- tetrads). The metallophilic attraction between the Na^+ ions is indicated by red dashed line. (**C**) and (**D**) Coordination of the Na^+ ions in-between the G- and T- tetrads (or T- and G- tetrads). The DNAs are shown as cartoon-and-stick views in atomic color (C, white; N, blue; O, red; P, orange), the Na^+ ions are shown as spheres in yellow.

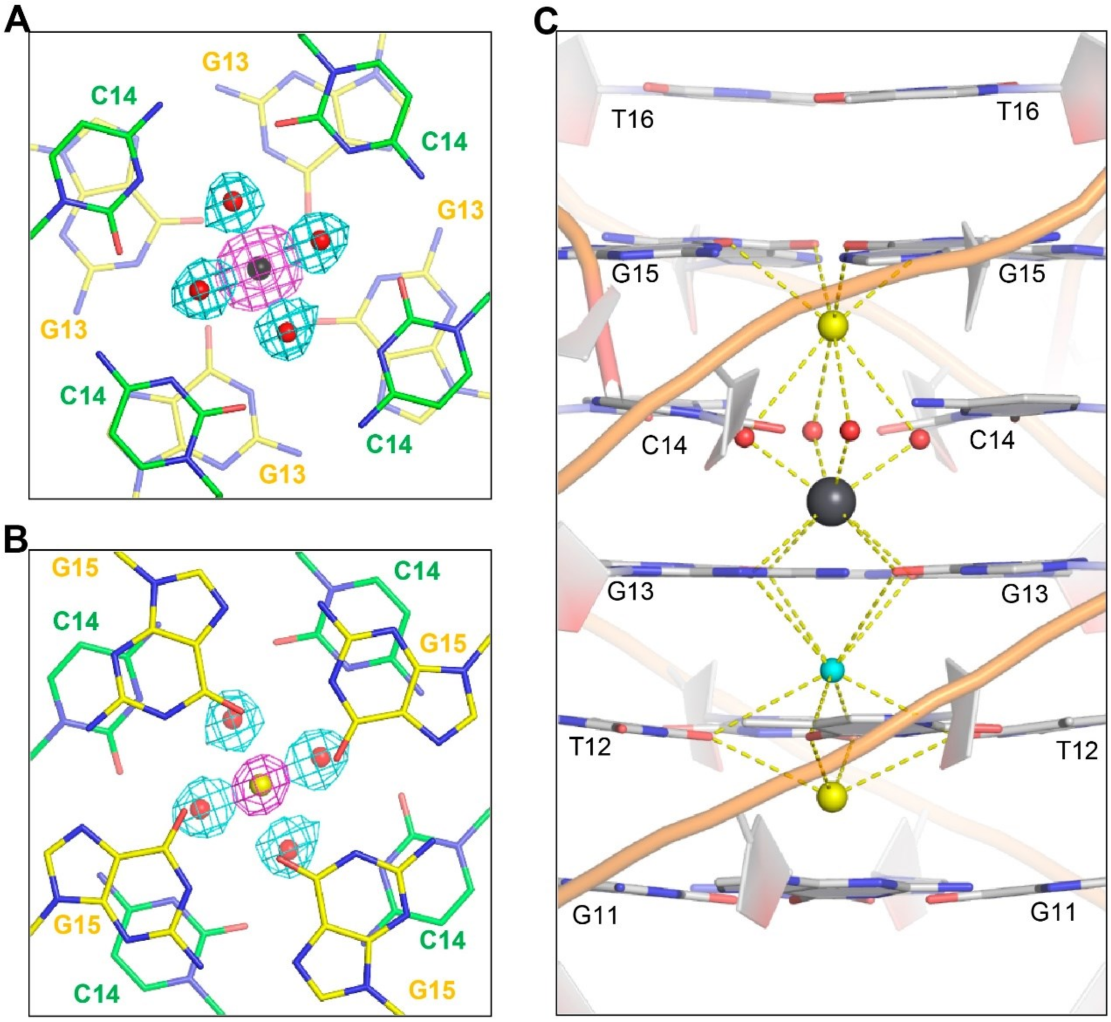

Supplementary Figure S6. Pb^{2+} ion recognition by DNA1. (**A**) A close view showing the Pb^{2+} ion bound in-between the G13-tetrad and C14-tetrad. (**B**) A close view showing the Na^{+} ion bound in-between the C14-tetrad and G15-tetrad. (**C**) Coordination of the cations bound at the central channel of the DNA1 structure. The Li^{+}, Na^{+}, Pb^{2+}, and the coordinating water molecules are shown as spheres in cyan, yellow, black, and red, respectively. The $2F_o$-F_c electron density maps of the Na^{+}, Pb^{2+}, and water molecules are all contoured at 1.5 sigma level in panels (**A**) and (**B**).

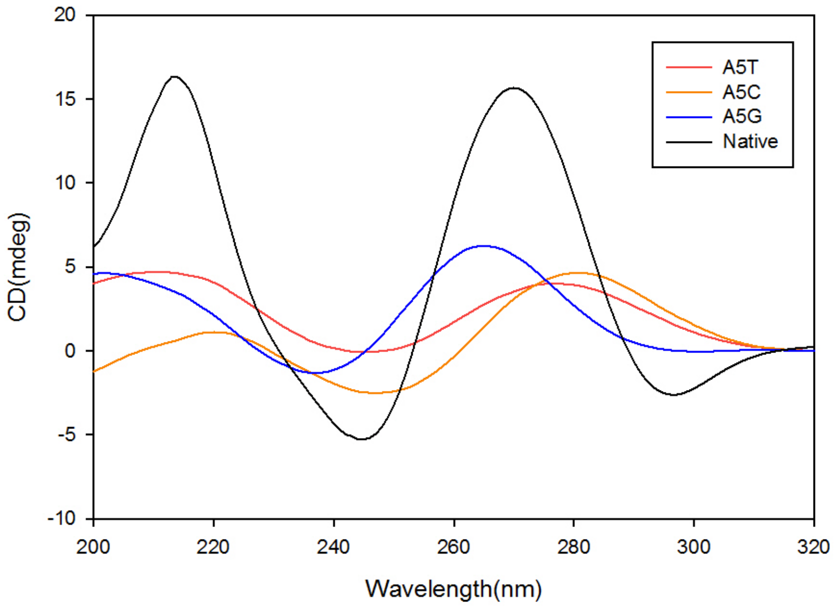

Supplementary Figure S7. CD analysis of the native and mutated DNA1 sequences with A5 mutated to T, C, G respectively. The x-axis is wavelength (nm) and the y-axis is CD absorption Δε ($M^{-1}cm^{-1}$).

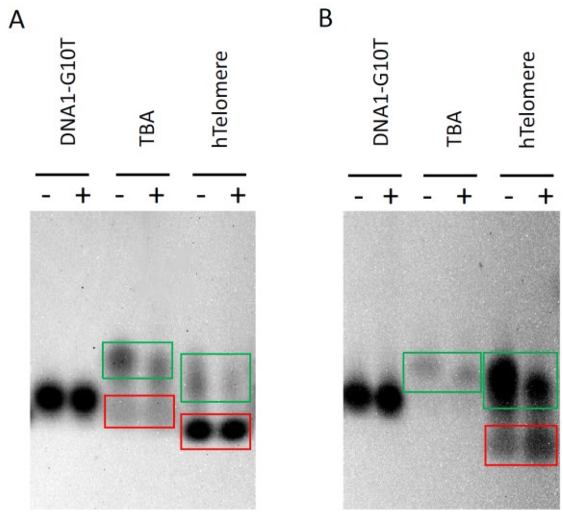

Supplementary Figure S8. Native gel analysis of TBA and hTelomere sequences. The single-stranded and quadruplex structures of TBA or hTelomere are highlighted with green and red boxes, respectively. In addition to TBE buffer pH 8.3, the native gel also contains 50 mM KCl in (**A**). No KCl is present in the native gel in (**B**). The denatured and annealed samples are indicated by “–” and “+” labels, respectively.

Supplementary Table S1. Data collection and structural refinement statistics.

	DNA1 (6A85)
Wavelength (Å)	0.97928
Space group	$P2_12_12$
Cell parameters	
a, b, c (Å)	37.8, 46.7, 51.3
α, β, γ (°)	90.0, 90.0, 90.0
Resolution rang (Å)	30.0-1.45
Outer shell (Å)	1.50-1.45
Completeness (%) [α]	96.1(87.3)
R_{sym} (%) [α]	7.6(26.2)
I/σ(I) [α]	64.8(12.1)
Redundancy [α]	12.8(11.5)
Resolution	25.5-1.45
No. of reflections	28779
R_{work} (%)	13.7
R_{free} (%)	16.9
r.m.s.d. bonds (Å)	0.009
r.m.s.d. angles (°)	1.169

[a]: Values in parentheses are for the outer shell.

Supplementary Table S2. Sequence of the native and mutant DNAs

Name	Sequence (from 5' to 3')
DNA1	AGAGAGATGGGTGCGTT
DNA1-del-5’-A	**-**GAGAGATGGGTGCGTT
DNA1-del-3’-TT	AGAGAGATGGGTGCG**--**
DNA1-del-A7	AGAGAG**-**TGGGTGCGTT
DNA1-delA7/G10A	AGAGAG**-**TG**A**GTGCGTT
DNA1-delA7/G10C	AGAGAG**-**TG**C**GTGCGTT
DNA1-delA7/G10T	AGAGAG**-**TG**T**GTGCGTT
DNA1-A5G	AGAG**G**GATGGGTGCGTT
DNA1-A5C	AGAG**C**GATGGGTGCGTT
DNA1-A5T	AGAG**T**GATGGGTGCGTT
DNA1-A7G	AGAGAG**G**TGGGTGCGTT
DNA1-A7C	AGAGAG**C**TGGGTGCGTT
DNA1-A7T	AGAGAG**T**TGGGTGCGTT
DNA1-T8G	AGAGAGA**G**GGGTGCGTT
DNA1-T8C	AGAGAGA**C**GGGTGCGTT
DNA1-T8A	AGAGAGA**A**GGGTGCGTT
DNA1-G9C	AGAGAGAT**C**GGTGCGTT
DNA1-G9A	AGAGAGAT**A**GGTGCGTT
DNA1-G9T	AGAGAGAT**T**GGTGCGTT
DNA1-G10C	AGAGAGATG**C**GTGCGTT
DNA1-G10A	AGAGAGATG**A**GTGCGTT
DNA1-G10T	AGAGAGATG**T**GTGCGTT
DNA1-G11C	AGAGAGATGG**C**TGCGTT
DNA1-G11A	AGAGAGATGG**A**TGCGTT
DNA1-G11T	AGAGAGATGG**T**TGCGTT
DNA Marker M1	GCTGCGCCTGGATGATC
